# Association between triglyceride–glucose index (TyG) as a surrogate marker of insulin resistance and risk of gestational diabetes mellitus (GDM): updated systematic review and meta-analysis

**DOI:** 10.61622/rbgo/2026rbgo47

**Published:** 2026-10-13

**Authors:** Gustavo Adolfo Vásquez-Tirado, Diego Alexis Moncada-Gutiérrez, Angie Farfán-Castillo, Edinson Dante Meregildo-Rodríguez, María Cuadra-Campos, Claudia Vanessa Quispe-Castañeda, Víctor Serna-Alarcón, Milene Villarreal-Jara

**Affiliations:** 1 Universidad Privada Antenor Orrego Facultad de Medicina Humana Trujillo Peru Facultad de Medicina Humana, Universidad Privada Antenor Orrego, Trujillo, Peru; 2 Universidad Peruana Cayetano Heredia School of Public Health and Administration Lima Perú School of Public Health and Administration, Universidad Peruana Cayetano Heredia, Lima, Perú; 3 Hospital Nacional Dos de Mayo Lima Perú Hospital Nacional Dos de Mayo, Lima, Perú

**Keywords:** Triglyceride-glucose index, Gestational diabetes *mellitus*, Insulin resistance, Risks

## Abstract

**Objective:**

This systematic review and meta-analysis evaluated whether the first-trimester triglyceride–glucose (TyG) index predicts subsequent gestational diabetes mellitus (GDM).

**Methods:**

MEDLINE/PubMed, Scopus, Embase, Web of Science, ScienceDirect, and the Cochrane Library were searched from inception to August 12, 2025. Eligible studies were prospective or retrospective cohorts including adult women with singleton pregnancies, first-trimester TyG assessment, and later GDM ascertainment; non-original studies, case reports/series, and duplicates were excluded. Risk of bias was assessed using ROBINS-E. Adjusted odds ratios (ORs) were pooled using random-effects models with REML and Hartung–Knapp adjustment. Sensitivity, subgroup, diagnostic accuracy, publication-bias, and GRADE certainty analyses were performed.

**Results:**

Fourteen observational cohorts comprising 462,233 pregnant women were included; 30,875 (7%) developed GDM. Twelve studies contributed adjusted association estimates. Elevated first-trimester TyG was associated with higher odds of GDM (OR 2.35, 95% CI 1.88–2.94; I^2^=92%). The association persisted after excluding influential studies (OR 2.36, 95% CI 1.91–2.90; I^2^=30.6%) and was consistent in Asian populations and low-risk-of-bias studies. Diagnostic performance was moderate, with pooled sensitivity of 0.62 (95% CI 0.54–0.69), specificity of 0.64 (95% CI 0.60–0.69), and AUC of 0.67. Limitations included substantial heterogeneity, variable TyG thresholds, observational designs, and limited non-Asian evidence.

**Conclusions:**

First-trimester TyG is associated with increased GDM risk and may support early risk stratification, but its moderate diagnostic performance precludes use as a standalone screening test.

**PROSPERO registry**: # CRD420251125511

## Introduction

Gestational diabetes mellitus (GDM) is one of the most common metabolic complications during pregnancy, with a global prevalence of approximately 14%.^(1)^ It is associated with maternal complications and adverse fetal outcomes, such as macrosomia, congenital malformations, and fetal death.^(2-4)^

GDM results from the interaction of genetic, physiological, and environmental factors that alter glucose homeostasis during gestation. Physiologically, pregnancy induces a transient state of insulin resistance and hyperglycemia; however, when this response becomes exacerbated, it contributes to placental dysfunction, generating a vicious cycle of metabolic dysregulation.^(5)^ Insulin plays a central role in regulating glucose metabolism and maintaining homeostasis.^(6)^ Consequently, reduced insulin sensitivity leads to pathological imbalance with perinatal repercussions. Furthermore, β-cell dysfunction secondary to chronic hyperglycemia further aggravates this process.^(7)^

Screening for GDM currently relies on the oral glucose tolerance test (OGTT), performed between 24 and 28 weeks of gestation.^(8)^ However, this approach has important clinical and logistical limitations.^(9)^ These limitations have prompted the search for alternative, simple, and early-applicable biomarkers.

Among the proposed biomarkers, the triglyceride-glucose (TyG) index has emerged as a cost-effective and easily obtainable surrogate marker of insulin resistance, derived from routine fasting triglyceride and glucose measurements.^(6,10)^ It is also considered a reliable indirect indicator of insulin resistance, with a sensitivity of 80% and specificity of 81%.^(11)^

Although fews systematic reviews and meta-analyses have explored the association between the TyG index and the risk of GDM, most have included pregnant women evaluated at diference trimesters,^(12,13)^ resulting in heterogeneus finding.

To date, no systematic review and meta-analysis has specifically evaluated the predictive performance of the TyG index measured exclusively in the first trimester of pregnancy for the subsequent development of GDM. Therefore, the present study aims to evaluate high-quality observational studies that analyze the associated between the first-trimester TyG index and its potential utility as an early risk indicator for GDM.

## Methods

### Registration, Search Strategy and Data collection

Our systematic review adheres to the methodological standards outlined in the Cochrane Handbook for Systematic Reviews,^(14)^ PRISMA guidelines,^(15)^ and AMSTAR 2 criteria.^(16)^ The protocol was prospectively registered in PROSPERO (CRD420251125511). We performed a comprehensive search of MEDLINE (via PubMed), Scopus, Embase, Web of Science, ScienceDirect, and the Cochrane Library. The strategy combined controlled vocabulary (e.g., MeSH, Emtree) and free-text terms using Boolean operators, aligned with our prespecified PECOS framework (Population: pregnant women; Exposure: first-trimester elevated triglyceride–glucose (TyG) index; Outcome: gestational diabetes mellitus; Study design: observational studies). Keywords were selected to capture the exposure ("triglyceride–glucose index") and the main outcome ("gestational diabetes mellitus"). The full search strings are provided in the Supplementary Material (Table 1S). All records retrieved during the primary and secondary screening phases were managed in Zotero (version 6.0.15). After duplication removal, the dataset was imported into Rayyan for screening. Two reviewers independently and blindedly screened titles and abstracts. Disagreements were resolved by consensus; when consensus could not be reached, a third reviewer adjudicated. Studies meeting inclusion criteria then underwent full-text assessment for eligibility. We also manually searched the reference lists and citation networks of included studies to identify additional records. The study selection process is summarized in figure 1. The full-text reviewed studies that were excluded are shown in Supplementary Material (Table 2S).

**Figure 1:**
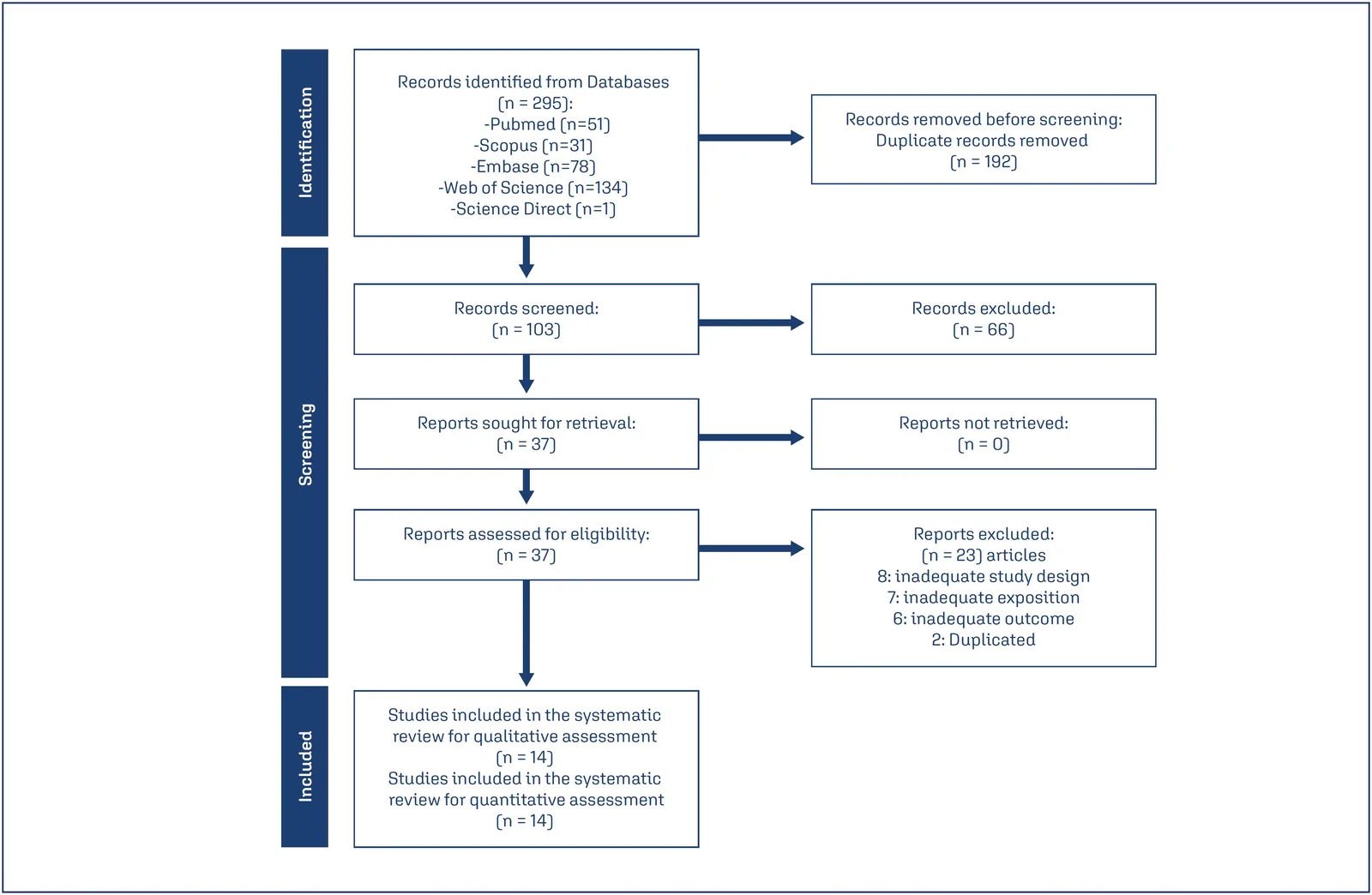
PRISMA flow diagram of the selection process of the primary studies included

### Eligibility criteria

We included observational cohort studies (prospective or retrospective) that evaluated the association between first-trimester TyG index and the subsequent risk of developing GDM. Eligible studies enrolled pregnant individuals aged ≥18 years with a singleton pregnancy. We imposed no restrictions on language or publication date and considered articles published through August 12, 2025. We excluded case reports/series, non-original articles (e.g., reviews, editorials), and duplicate publications.

### Data collection process and data items

Two independent researchers collected and extracted relevant information from each included study using a standardized, blinded spreadsheet. This information included authors’ names, country and year of publication, study design, clinical and epidemiological characteristics of the population, number of participants and cases, TyG index cut-off points, measures of association, confounding factors, and outcomes.

### Data items

GDM: The included articles defined GDM according to the International Association of Diabetes and Pregnancy Study Groups^(27)^ (IADPSG) criteria, using a 75 g oral glucose tolerance test between 24 and 28 weeks of gestation. The diagnostic thresholds are 92 mg/dL, 180 mg/dL and 153 mg/dL at baseline, 1 hour and 2 hours after the OGTT, respectively.

TyG: The included studies considered the TyG index as a variable to determine the development of GDM in the second or third trimester of pregnancy. Additionally, in all studies the TyG index was calculated using triglyceride and fasting plasma glucose values through the following formula: ln (triglyceride (mg/dL) x fasting plasma glucose (mg/dL)/2),^(28)^ it should be emphasized that the index itself, and its interpretation, are unitless. In the absence of standardized and universally accepted TyG cutoff values, particularly in the context of GDM, and given the heterogeneity of the thresholds used across the included studies, most studies assessed the TyG index by categorizing it into tertiles, quartiles, or quintiles and then estimating measures of association for each category. In the present study, the values corresponding to the highest tertile or quartile were selected.

### Statistical analysis

We employed the Inverse Variance (IV) method^(19-21)^ in the meta-analysis to combine adjusted odds ratios (ORs) and their 95% confidence intervals (CIs). This analysis was performed using R® 4.2.226 software. For dichotomous variables, we calculated odds ratios (OR) with 95% confidence intervals (CI 95%) based on the events in exposed and non-exposed groups. Missing data were reported when appropriate. For continuous variables, we gathered the mean and standard deviation (SD) for both exposed and non-exposed groups. When studies presented these variables as medians and interquartile ranges (IQR), we converted them to means and SDs using appropriate methods.^(22,23)^

Forest plots were used to summarize the quantitative synthesis, utilizing the *meta* library, and the *metabin*^(21-23)^ (for dichotomous variables) and *metacont*^(23)^ (for continuous variables) functions. The IV method with Restricted Maximum Likelihood (REML) for tau^2^ was applied. Heterogeneity among studies was assessed using Cochran's Q test and Higgins I^2^ statistic. In cases of statistically significant heterogeneity (I^2^ statistics > 40%), a random effects model with Hartung-Knapp (HK) adjustment was employed. Sensitivity analysis was conducted using Influence Analysis with the *InfluenceAnalysis* function, and Graphic Display of Heterogeneity (GOSH) with the *gosh.diagnostics* function.^(24)^ Additionally, due to the variability in the measures of association for continuous variables across studies, their inclusion in the meta-analysis was deemed inappropriate.

### Sensitivity analysis

A sensitivity analysis was performed when significant heterogeneity (I^2^ > 40%) was identified. This analysis included Influence Analysis using the *InfluenceAnalysis* function^(25)^ and a graphic display of heterogeneity (GOSH)^(22,26)^ using the *gosh.diagnostics* function in R version 4.2.2.

### Quality assessment

The Risk of Bias in Non-Randomized Studies–of Exposures (ROBINS-E)^(29)^ tool was used to evaluate each study individually for the potential risk of bias, given that this investigation included observational studies. Furthermore, a funnel plot was generated, and Egger's test was conducted to evaluate the potential presence of publication bias. The quality of evidence for all outcomes was evaluated using the Grading of Recommendations Assessment, Development and Evaluation (GRADE) criteria. The resolution of disagreements among the reviewers was achieved through discussion with the principal investigator.

## Results

### Search results and study characteristics

Fourteen primary studies were included in the qualitative and quantitative synthesis.^(30-43)^ Of these, twelve reported the association between the TyG index and GDM using odds ratios (ORs) as the effect measure,^(30-32,34-37,39-43)^ and twelve provided data on the predictive performance of TyG, including sensitivity, specificity, and area under the curve (AUC);^(30-36,38-42)^ several studies reported both types of outcomes. Regarding study design, nine were prospective cohort studies^(30-32,34,36,38-40,42)^ and five were retrospective cohort studies.^(33,35,37,41,43)^ Eight studies were conducted in Chinese^(30-33,35-37,43)^ populations, three in Mexico,^(38,39,42)^ two in Korea^(34,41)^ and one in Iran,^(40)^ comprising a total of 462,233 pregnant women, of whom 30,875 (7%) developed GDM. All included studies measured the TyG index during the first trimester of pregnancy, whereas the diagnosis or assessment of GDM risk was performed in the second trimester. Some studies also evaluated pregnancy-related complications and adverse perinatal outcomes, including gestational hypertension, premature rupture of membranes, preterm delivery, large and small for gestational age (LGA/SGA) newborns, fetal distress, and other related events. Additional demographic characteristics of the study population are detailed in chart 1.

**Chart 1:** General characteristics of included studies

Author	Study design	Participants	TyG values	Exposition/comparator	Outcome	Association measures	Confounding adjustment
Guo Y et al.^(30)^ 2024. China	PC	1624 women with singleton pregnancies > 18 years old and < 14 weeks of gestation were enrolled. Data were collected from the Northwest China Project Site between August 2021 and April 2023. The average age of the pregnant women was 31.7 years and 27.52% of women developed GDM. For each 1-unit rise in the TyG index, the risk of GDM increased 2.10-fold and the risk of gestational hypertension increased by 67%.	Quintiles: Q1: TyG index ≤ 8.273 (GDM: 14.7%). Q2: TyG index - (GDM: 18.0%) Q3: TyG index - (GDM: 22.2%). Q4: TyG index - (GDM: 28.0%). Q5: TyG index ≥ 9.294 (GDM: 39.4%). Quintile 5 was associated with a 3.25-fold increase in the risk of GDM compared with the lowest quintile group (Q1).	TyG index > 8.890	Primary outcome: GDM Secondary outcome: Other PRCs including GH.	Adjusted OR: 3.25 (95% CI: 2.23–4.75) . GDM prediction 2 x 2 table: TP (275), FP (451) FN (172), TN (726)	Maternal age, pre-pregnancy BMI, ethnicity, assisted reproduction, miscarriage history, gravidity, parity, gestational age at delivery, GWG, SBP, DBP, LDL-C, HDL-C, Hb, UA, ALT and AST, TC and Cr.
Li H et al.^(31)^ 2022. China	PC	Data were obtained from the Fujian Birth Cohort Study (FJBCS), which included 11 387 singleton pregnant women > 18 years of age and < 14 weeks of gestation. Among them, 43.0% were the first gravidity and 24,48% developed GDM. The average age was 30.0 ± 4.0 years. The risk of GDM increased 3.63-fold for each 1-unit increase in the TyG index and 1.57-fold for each 1-SD increase, respectively.	Quintiles: Q1: TyG index 7.36-8.19 (GDM: 16.3%). Q2: TyG index 8.20-8.38 (GDM: 20.9%) Q3: TyG index 8.38-8.55 (GDM: 24,6%). Q4: TyG index 8.55-8.76 (GDM: 33,2%). Q5: TyG index 8.76-9.89 (GDM: 42,6%). The group with the highest TyG index (Q5) had a 3.14-fold higher risk of GDM than the group in the lowest quintile (Q1).	TyG index > 8.549	Primary outcome: GDM, PRCs such as GH, PE, and premature rupture of membranes.	Adjusted OR: 3.14 (95% CI: 2.55 – 3.85). GDM prediction 2 x 2 table: TP (1491), FP (2761) FN (1296), TN (5839).	Pregnancy BMI, maternal age, assisted reproduction, abortion history, gravidity, parity, GWG, delivery gestations, gestational week at the examination, SBP, DBP, TC, LDL-C, HDL-C, Hb, UA, Cr, ALT and AST.
Liu P et al.^(32)^ 2020. China	PC	This research was conducted in a single center between October and December 2018. After excluding women with multiple pregnancies and those with irrelevant variables, 352 female patients were evaluated. The overall incidence of GDM was 18.75%. The average age was 29.2 years and it was higher in the GDM group (30.4 vs 29.0). The prevalence of LGA differed significantly between women with and without GDM.	Tertiles: Distribution of GDM cases by TyG index: Tertile 1: TyG index < 7.9 (GDM: n=10). Tertile 2: TyG index 7.9–8,3 (GDM: n=19). Tertile 3: TyG index ≥ 8.3 (GDM: n=37). Women in the highest tertile (T3) had a significantly higher risk of developing GDM.	TyG index > 8.3	Primary outcome: GDM, LGA newborn.	Adjusted OR: 3.54 (95% CI: 1.48 – 8.43). GDM prediction 2 x 2 table: TP (37), FP (92) FN (29), TN (194).	Age, gestation age, BMI at enrolment, GWG, education, physical activity, parity, family history of diabetes, history of PCOS, CRP, mode of delivery.
Ma N et al.^(33)^ 2024. China	RC	A total of 1073 adult women with singleton pregnancies were enrolled between June 2017 and September 2019. Exclusion criteria: Diseases affecting blood glucose levels, including hyperthyroidism, Cushing's syndrome, and pancreatitis. 18.7% of participants developed GDM. In the GDM group, age was higher compared to the NGT group (29.21 ± 3.81 vs. 27.97 ± 3.65) and had higher levels of GGT, CREA, UA, glycated hemoglobin, TG, and LDL-C during the first trimester of pregnancy. TyG index: 7.23 ± 0.37 in The GDM group, 6.99 ± 0.36 in the NGT group.	(p < 0.001): The GDM group: TyG index 7.23 ± 0.37 The NGT group: TyG index 6.99 ± 0.36	TyG index > 7.08	Primary outcome: GDM	Adjusted OR: Not reported. GDM prediction 2 x 2 table: TP (137), FP (312) TN (64), VN (560).	Not reported
Mo Z et al.^(34)^ 2024. Korea	PC	589 pregnant women who were carrying a single fetus were evaluated. The primary data used are publicly available. The mean age was 32.065 ± 3.798 years. The incidence of GDM was 6,11%. The TyG index (8.352 ± 0.400) was positively correlated with the development of GDM. An inverse association was observed between HDL-C and the development of GDM.	Tertiles: Distribution of GDM cases by TyG index: Tertile 1: TyG index ≤ 8.181 (GDM: n=2). Tertile 2: TyG index 8.181 ≤ 8.514 (GDM: n=6). Tertile 3: TyG index > 8.514 (GDM: n=28). Women in the Tertile 3 group tended to be older and have higher levels of insulin and fasting glucose.	TyG index > 8.632	Primary outcome: GDM	Adjusted OR: 5.586 (95% CI: 1.15 – 26.92). GDM prediction 2 x 2 table: TP (25), FP (118) FN (11), TN (435).	Age, pre-pregnancy BMI, hepatic steatosis, parity, ALT, AST, GGT, TC, HDL-c, insulin.
Zhang J et al.^(35)^ 2025. China	RC	This study was conducted in a single center between October and December 2018. A total of 631 pregnant women at 5-8 weeks of gestation were enrolled; of which 16.2% developed GDM and the majority of them were < 35 years. The analysis showed that a 1-unit rise in the TyG index corresponded to a 2.3-fold higher risk of GDM.	Quartiles: Distribution of GDM cases by TyG index: Q1: GDM: 7% Q2: GDM: 12.1% Q3: GDM: 17.1% Q4: GDM: 28.5% The risk of GDM increased steadily with each quartile of the TyG index.	TyG index > 8.06	Primary outcome: GDM, PD, HDP, ICP, LGA and premature rupture of membranes.	Adjusted OR: 2.26 (95% CI: 1.27 – 4.02). GDM prediction 2 x 2 table: TP (82), FP (268) FN (20), TN (261).	Age, BMI, SBP, DBP, history of miscarriage, delivery, TC, UA, LDL, TBIL, and HDL.
Zhang L et al.^(36)^ 2025. China	PC	46992 women with singleton pregnancies, > 18 years old, between 6 and 13 weeks of gestation were enrolled from the Beijing Birth Cohort Study (BBCS), between 2018 and 2022. The incidence of GDM was 12.86%, of which the average age was 34.52 ± 3.97. TyG index: 8.39 ± 0.41 in the GDM group, 8.18 ± 0.38 in the non-GDM group. This study demonstrated that a per unit increase in the TyG index during early pregnancy is linked to a 1.80-fold increase in the risk of GDM.	Quartiles: Q1: <7.94 Q2: ≥ 7.94, <8.18 Q3: ≥ 8.18, <8.45 Q4: ≥ 8.45 The odds of developing GDM were 1.52 times higher in Q1, 2.18 times higher in Q2, and 3.87 times higher in Q3 and Q4.	TyG index > 8.30	Primary outcome: GDM	Adjusted OR: 2.8 (95% CI: 2.58 – 3.03). GDM prediction 2 x 2 table: TP (3627), FP (14741) FN (2418), TN (26206).	Age, primiparity, education level, pre-pregnancy BMI, history of GDM, family history of diabetes and first-trimester LDL-C and CHO levels.
Song S et al.^(37)^ 2025. China	RC	11 942 pregnant women ≥18 years and < 14 weeks of pregnancy were enrolled between May 2019 and April 2024, of which 15.4% developed GDM. The average maternal age was 30.12 ± 4.09 years and the fasting glucose level was 84.16 ± 9.27. The overall average TyG index was 8.36 ± 0.41, while the average TyG index in Q4 was 8.89 ± 0.26.	Quartiles: Q1: TyG index 6.14–8.08 (GDM: 10.3%). Q2: TyG index 8.09–8.33 (GDM: 12.8%) Q3: TyG index 8.34–8.61 (GDM: 15.4%). Q4: TyG index 8.62–10.72 (GDM: 23.2%). The result was significantly different in all quartiles: For each one-unit increase in the TyG index, the risk of GDM increased by 2,21-fold.	TyG index: cutoff not reported.	Primary outcome: GDM, PE, GH, PD	Adjusted OR: 2.21 (95% CI: 1.9 – 2.57)	Age, Pre-pregnancy, education, BMI, Gravidity, gestational week at examination, Parity, DBP, SBP, TC, HDL, LDL, HbA1c, ALB and PT.
Sánchez-García A et al.^(38)^ 2020. México	PC	140 pregnant women ≥18 and ≤40 years of age were enrolled from the antenatal care clinic at the Obstetric Division, between June 2018 and December 2018. The incidence of GDM was 27.1%. The women with GDM had an average maternal age of 24.7 ± 6.2 years, and was similar in both groups. Plasma glucose levels increased by 33.3% after one hour and by 35.9% after two hours. The TyG index was 4.88 ± 0.70 in the GDM group and 4.68 ± 0.19 in the non-GDM group.	Tertiles: GDM cases according to the distribution of the TyG index: Tertile 1: 2/47. Tertile 2: 12/46. Tertile 3: 24/47. Tertile 3 was associated with a five-fold increase in the risk of GDM compared with those in the lowest tertile (T1).	TyG index > 4.69	Primary outcome: GDM. Secondary outcome: PE. PD, LGA, Fetal distress, Hypoglycemia, among others.	Adjusted OR: Not reported. GDM prediction 2 x 2 table: TP (34), FP (51) FN (4), TN (51)	Not reported.
Sánchez-García A et al.^(39)^ 2020. Mexico	PC	164 pregnant women ≥18 years of age and <14 weeks of pregnancy were enrolled. This study was performed at a single center from November 2017 to October 2019. The incidence of GDM was 17.7%. The women with GDM had an average maternal age of 24.9 ± 5.4 years. Women who showed 1, 2, or 3 abnormal glucose values had corresponding proportions of 75.9%, 20.7%, and 3.4%, respectively. TyG index in the first trimester was not significant, but it was in the second trimester evaluation: TyG index: 9.01 ± 0.3 in the GDM group and 8.73 ± 0.34 in the non-GDM group.	Tertiles: Distribution of GDM cases by TyG index: Tertile 1: TyG index ≤8.24 (GDM: 16.4%). Tertile 2: TyG index 8.25–8.56 (GDM: 21.2%). Tertile 3: TyG index ≥8.57 (GDM: 15.8%). There were no significant differences in TyG index values regarding the development of GDM.	TyG index > 8.7	Primary outcome: GDM. Secondary outcome: hyperbilirubinemia, LGA, SGA, among others.	Adjusted RR: 1.03 (95% CI: 0.57–1.88). GDM prediction 2 x 2 table: TP (9), FP (48) FN (20), TN (87)	DBP, parity, family history of diabetes and first trimester BMI.
Pazhohan A et al.^(40)^ 2017. Iran	PC	A total of 954 women with singleton pregnancies aged 18 to 35 years agreed to participate in the study. This study was conducted in at six health centers, between August 2014 and February 2016. GDM was identified in 18.4% of the patients. The women with GDM had an average maternal age of 27.47 ± 3.54 years. Compared with the control group, the GDM group showed a significantly higher mean fasting plasma glucose level (91.3 ± 8.7 vs. 82.9 ± 7.0). TyG index: 9.31 ± 6.13 in the GDM group and 8.34 ± 5.04 in the control group.	Tertiles: Tertile 1: TyG index <8.31. Tertile 2: TyG index: 8.31–8.98. Tertile 3: TyG index ≥ 8.99. Tertile 3 was associated with a 4.9-fold increase in the risk of GDM compared with those in the lowest tertile.	TyG index ≥ 8.99	Primary outcome: GDM, LGA.	Adjusted RR: 4.91 (95% CI: 1.74–13.69). GDM prediction 2 x 2 table: TP (97), FP (218) FN (79), TN (560).	Age, history of diabetes in the first-degree family and first trimester-body mass index.
Kim J et al.^(41)^ 2020. Korea	RC	A total of 380 208 pregnant women were included. The present investigation used the NHIS database, covering data from January 2012 to December 2015. The participants were primiparous. The incidence of GDM was 4.53%. The women included in Q4 had an average maternal age of 31.05 ± 3.63 years. The TyG index and screening examinations were measured within 2 years before first delivery. The risk of GDM increased 1.81 times for each 1-unit increase in the TyG index and 31% for each 1-SD increase respectively.	Quartiles: Q1: TyG index 7.44 Q2: TyG index 7.81 Q3: TyG index 8.09 Q4: TyG index 8.59 Q4 was associated with a 1.73-fold increase in the risk of GDM compared with Q1, Q2 and Q3. The risk of GDM requiring insulin therapy was higher in the fourth quartile (Q4) of the TyG index.	GDM: TyG index > 8.15 GDM without insulin treatment: TyG index > 8.12 GDM with insulin treatment: TyG index > 8.26	Primary outcome: GDM, Secondary outcome: GDM without insulin therapy and GDM with insulin therapy.	Adjusted OR: 1.73 (95% CI: 1.65-1.81). GDM prediction 2 x 2 table: TP (8 102), FP (115 424) FN (9 137), TN (247 545).	Age, BMI, heavy alcohol consumption, smoking status, a family history of diabetes and regular exercise.
Gurza G et al.^(42)^ 2025. Mexico	PC	A total of 333 women were included in OBESO cohort between 2017 and 2019. Women were ≥18 years, with singleton pregnancies < 15 weeks of gestation. Group 1 (TyG index > 8.6) had a higher average maternal age compared to Group 2 (TyG index ≤ 8.6): 30.8 ± 5.1 vs. 28.9 ± 5.4. GDM was diagnosed in 22.9% of pregnant women in group 1 and in 11.1% of pregnant women in group 2. No significant association was reported between a high TyG index and other APOs with the exception of preeclampsia.	Percentiles: 25th percentile: TyG index 8.0. 50th percentile: TyG index 8.3. 75th percentile TyG index 8.6. High TyG index > 8.6 was associated with a 1.87-fold increase in the risk of GDM.	TyG index > 8.6	Primary outcome: GDM, PD, cesarean section, HDP, PE, SGA, LGA.	Adjusted RR: 1.87 (95% CI: 1.0–2.5). GDM prediction 2 x 2 table: TP (35), FP (118) FN (20), TN (160).	Maternal age, pregestational weight, and BMI.
Cui J et al.^(43)^ 2025. China	RC	Of the 11 477 women with singleton pregnancies who were enrolled, 8605 were selected for the study after applying the inclusion and exclusion criteria. This study was conducted in a single center, between 2015 and 2021. GDM was identified in 21.1% of the patients. The average maternal age was 30.0 ± 4.1 years. The TyG index was 8.39 ± 0.40 in all participants. The risk of GDM increased 1.35 times for each 1-SD increase of TyG index.	Percentiles: 25th percentile: TyG index 8.11. 50th percentile: TyG index 8.36. 75th percentile TyG index 8.63. Quartiles: Q1: Ref. Q2: OR 1.11 (0.94–1.31). Q3: OR 1.38 (1.17–1.63). Q4: OR 2.03 (1.73–2.39). Q4 was associated with a 2.03-fold increase in the risk of GDM compared with those in the lowest tertile.	TyG index: cutoff not reported.	Primary outcome: GDM, LBW, PD, hypertension, PE, SGA, LGA.	Adjusted OR: 2.03 (95% CI: 1.73–2.39).	Maternal age, education level, BMI, family history of diabetes and parity.

PC: Prospective Cohort; RC: Retrospective Cohort; GDM: Gestational diabetes mellitus; TyG index: Triglyceride-glucose index; TyG-BMI index: Triglyceride-Glucose Body Mass Index; PRCs: pregnancy-related complications; APOs: adverse perinatal outcomes; BMI: Body Mass Index; GWG: Gestational Weight Gain; LBW: Low birth weight; GH: Gestational Hypertension; PE: preeclampsia; PD: Preterm delivery; SBP: Systolic Blood Pressure; DBP: Diastolic Blood Pressure; LGA: Large for Gestational Age; SGA: Small for Gestational Age; PCOS: Polycystic Ovary Syndrome; CRP: C-reactive protein; NGT: Normal Glucose Tolerance; GGT: Gamma-Glutamyl Transferase; CREA: Creatinine; UA: Uric Acid; TG: Triglycerides; LDL-C: Low-Density Lipoprotein Cholesterol; HDL-C: High-Density Lipoprotein Cholesterol; PT: Prothrombin time; ALB: Albumin; OGTT: Oral Glucose Tolerance Test; OGTT 2hPG: Oral Glucose Tolerance Test 2-hour Plasma Glucose; FBG: fasting blood-glucose; HDP: Hypertensive Disorders of Pregnancy; PIH: Pregnancy-induced hypertension; ICP: Intrahepatic Cholestasis of Pregnancy; HTG: Hypertriglyceridemia; NAFLD: Nonalcoholic fatty liver disease; FFA: Free fatty acid; HOMA-IR: Homeostasis model assessment-insulin resistance; TC: total cholesterol; TBL: total bilirubin; CHO: cholesterol; TP: True Positives; FP: False Positives; FN: False Negatives; TN: True Negatives

### Risk of bias in studies

Risk of bias was assessed using the ROBINS-E tool for observational studies.^(29)^ This instrument evaluates observational evidence across seven key domains to determine the overall risk of bias: confounding, exposure measurement, selection of participants, post-exposure interventions, missing data, outcome measurement, and selection of the reported result. Based on this assessment, ten studies were judged to be at low risk of bias, one at some concerns, and four at high or very high risk of bias. For further details, see chart 2.

**Chart 2:** Risk of bias in the included studies using the ROBINS-E tool

Authors	Domain 1: Risk of Bias Due to Confounding	Domain 2: Risk of bias arising from measurement of the exposure	Domain 3: Risk of bias in selection of participants into the study (or into the analysis)	Domain 4: Risk of bias due to post-exposure interventions	Domain 5: Risk of bias due to missing data	Domain 6: Risk of bias arising from measurement of the outcome	Domain 7: Risk of Bias in Selection of the Reported Result	Conclusion
Guo Y et al.^(30)^	Low risk	Low risk	Low risk	Low risk	Low risk	Low risk	Low risk	Low risk
Li H et al.^(31)^	Low risk	Low risk	Low risk	Low risk	Low risk	Low risk	Low risk	Low risk
Liu P et al.^(32)^	Low risk	Low risk	Low risk	Low risk	Low risk	Low risk	Low risk	Low risk
Ma N et al.^(33)^	High risk	Low risk	Low risk	Low risk	Low risk	Low risk	High risk	High risk
Mo Z et al.^(34)^	Low risk	Low risk	Low risk	Low risk	Low risk	Low risk	Low risk	Low risk
Zhang J et al.^(35)^	Low risk	Low risk	Low risk	Low risk	Low risk	Low risk	Low risk	Low risk
Zhang L et al.^(36)^	Low risk	Low risk	Low risk	Low risk	Low risk	Low risk	Low risk	Low risk
Song S et al.^(37)^	Low risk	Low risk	Low risk	Low risk	Low risk	Low risk	Low risk	Low risk
Sánchez-García A et al.^(38)^	High risk	Low risk	Low risk	Low risk	Low risk	Low risk	Low risk	High risk
Sánchez-García A et al.^(39)^	Low risk	Low risk	Low risk	Low risk	Low risk	Low risk	Low risk	Low risk
Pazhohan A et al.^(40)^	Some concerns	Low risk	Low risk	Low risk	Low risk	Low risk	Low risk	Some concerns
Kim J et al.^(41)^	Low risk	Low risk	Low risk	Low risk	Low risk	Low risk	Low risk	Low risk
Gurza G et al.^(42)^	High risk	Some concerns	Low risk	Low risk	Low risk	Low risk	Low risk	High risk
Cui J et al.^(43)^	Low risk	Low risk	Low risk	Low risk	Low risk	Low risk	Low risk	Low risk

### TyG as a predictor of GDM

Among the twelve studies included in the meta-analysis that reported effect size estimates, we found that an elevated first-trimester TyG index was associated with approximately a 2.4-fold increase in the odds of developing GDM (pooled OR 2.35; 95% CI 1.88–2.94; I^2^ = 92%) compared with pregnant women with normal TyG values. Although heterogeneity was unacceptably high (I^2^ = 92%), the prediction interval (PI) remained above the null value, indicating that, even across different clinical settings, the exposure is likely to be associated with an increased risk of GDM (Figure 2A). When exploring the observed heterogeneity through sensitivity analyses with Influence Analysis, we found that the studies by Sánchez-García et al.^(39)^ and Kim et al.^[41]^ behaved as outliers. After excluding these studies, heterogeneity improved while the predictive effect on the risk of developing GDM was maintained (OR 2.59; 95% CI 2.19–3.07; I^2^ = 64.6%) (Figure 2B). Similarly, in the sensitivity analysis using the Graphic Display of Study Heterogeneity (GOSH), the studies by Li et al.,^(31)^ Zhang et al.,^(36)^ Sánchez-García et al.,^(39)^ and Kim et al.^(41)^ were identified as influential contributors to heterogeneity. Once these studies were excluded, we again observed that the association between elevated TyG and the risk of GDM persisted (OR 2.36; 95% CI 1.91–2.90; I^2^ = 30.6%), with an acceptable level of heterogeneity (Figure 2C).

**Figure 2:**
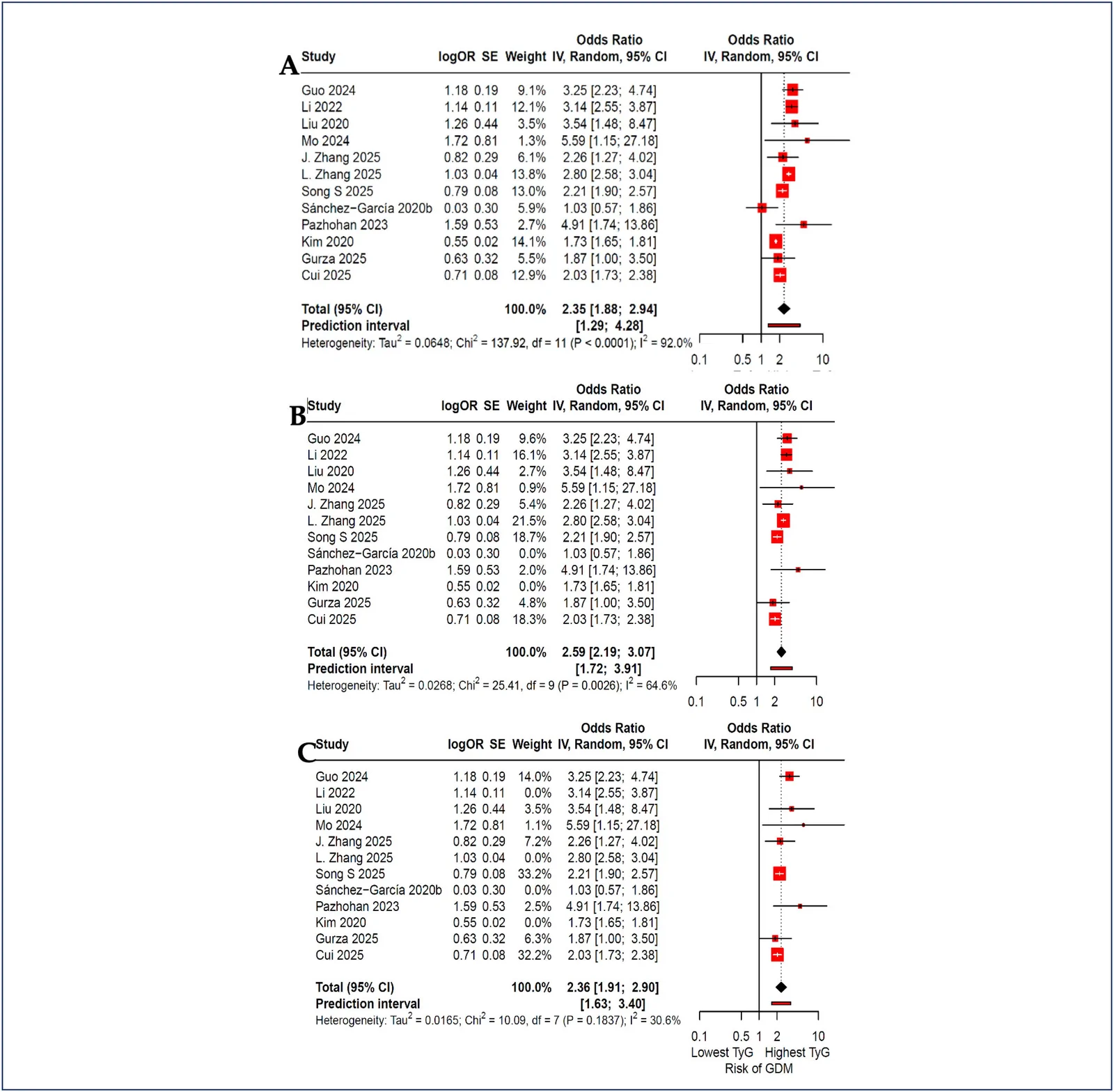
Forest plot of the association between the TyG index and the risk of GDM. All included studies were meta-analyzed (A), showing substantial heterogeneity; however, the prediction intervals remained acceptable. Sensitivity analysis using *Influence Analysis* (B) identified the studies by Sánchez-García and Kim as outliers, while the *GOSH analysis* (C) additionally highlighted the studies by Li, L. Zhang, and the afore mentioned outliers. Overall, a positive association between TyG and the risk of GDM was observed, with a tendency toward reduced heterogeneity after accounting for these influential studies

Conversely, when we evaluated the association between TyG and the risk of GDM in subgroup analyses by Asian versus non-Asian populations, we found that pregnant women of Asian origin with elevated first-trimester TyG had 2.64 times higher odds of developing GDM compared with those with normal TyG values (OR 2.64; 95% CI 2.21–3.15; I^2^ = 67.1%), with a modest reduction in heterogeneity. Comparison with the non-Asian subgroup is not possible, because after excluding Sánchez-García et al.,^(39)^ only the study by Gurza et al.^(42)^ remains (Figure 3A). In subgroup analyses according to risk of bias, we found that studies judged to be at low risk of bias showed 2.60 times higher odds of GDM (OR 2.60; 95% CI 2.18–3.11; I^2^ = 69.4%), whereas no statistically significant association was observed in studies with any concerns or high/very high risk of bias (OR 2.76; 95% CI 0.01–1137.45; I^2^ = 32.1%) (Figure 3B).

**Figure 3:**
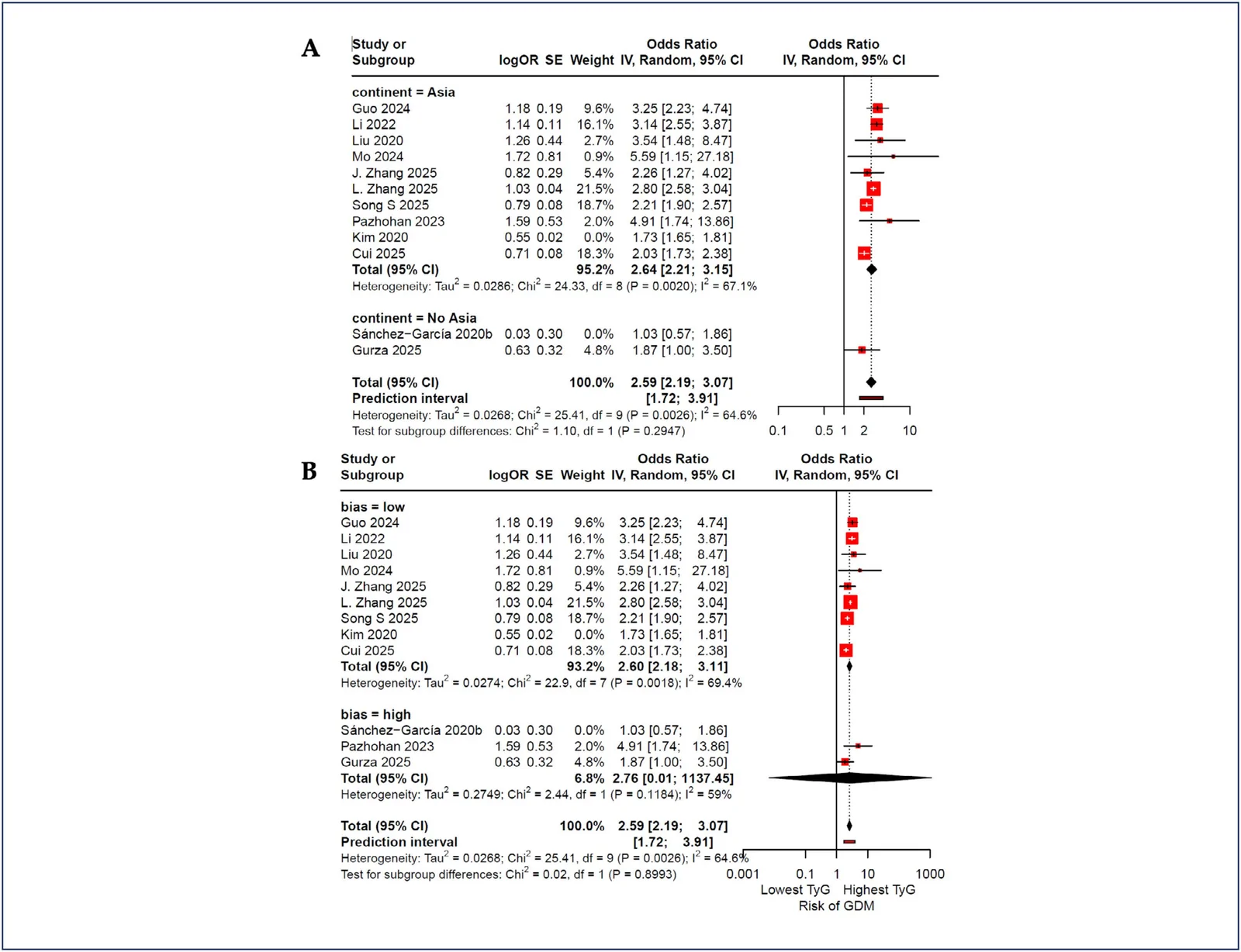
Forest plot of subgroup analyses by continent (Asian vs. non-Asian), showing that elevated TyG is associated with an increased risk of GDM only in the Asian population, with a moderate reduction in heterogeneity (A). In addition, subgroup analysis by risk-of-bias level showed a similar pattern among studies judged to be at low risk of bias. In both subgroup evaluations, the outlier studies by Sánchez-García and Kim were excluded

Given the limited number of studies and their higher clinical plausibility, we selected continent (Asia vs. non-Asia) and the TyG cut-off value as the main moderators. In mixed-effects meta-regression models (k = 13), these two moderators accounted for about one-fifth of the between-study heterogeneity (R^2^ = 21.02%), although substantial residual heterogeneity remained (residual I^2^ = 84.10%; τ^2^ = 0.0507; QE_10_ = 87.36; p < 0.001). The joint test of moderators did not reach statistical significance (F_2,10_ = 2.90; p = 0.10), indicating that, taken together, continent and TyG cut-off did not conclusively explain variability in effect sizes. When examined individually, studies conducted outside Asia tended to report smaller effects than those from Asian populations (β = −0.58; SE = 0.27; p = 0.056; OR ≈ 0.56), whereas higher TyG cut-off values were associated with a non-significant increase in the log odds of GDM (β = 0.56; SE = 0.34; p = 0.13; OR ≈ 1.76). Overall, these findings may indicate an attenuated association in non-Asian populations and a modest increase in risk at higher TyG thresholds. Nevertheless, because the statistical support is limited and substantial residual heterogeneity persists, the evidence remains imprecise and insufficient to draw firm conclusions regarding an association in non-Asian populations (Supplementary Material, Table 3S)

### Diagnostic performance of the TyG index as a predictor of GDM

Twelve of the included studies reported diagnostic accuracy metrics for TyG as a predictor of GDM, including sensitivity (Se), specificity (Sp), positive likelihood ratio (LR+), and negative likelihood ratio (LR−). When meta-analyzing these results, we found that TyG in predicting GDM has a pooled sensitivity of 0.62 (95% CI 0.54–0.69; I^2^: 97%), a pooled specificity of 0.64 (95% CI 0.60–0.69; I^2^: 97%), a pooled LR+ of 1.73 (95% CI 1.6 to 1.9), a pooled LR- of 0.6 (95% CI 0.51 to 0.69), a pooled diagnostic OR of 2.94(95% CI 2.0 to 4.3), and a false positive rate (FPR) of 0.36 (95% CI 0.31 to 0.40). The summary ROC (sROC) curve showed a moderate diagnostic performance, with an AUC of 0.67 (SE 0.0046, Q* 0.63) (Figure 4).

**Figure 4:**
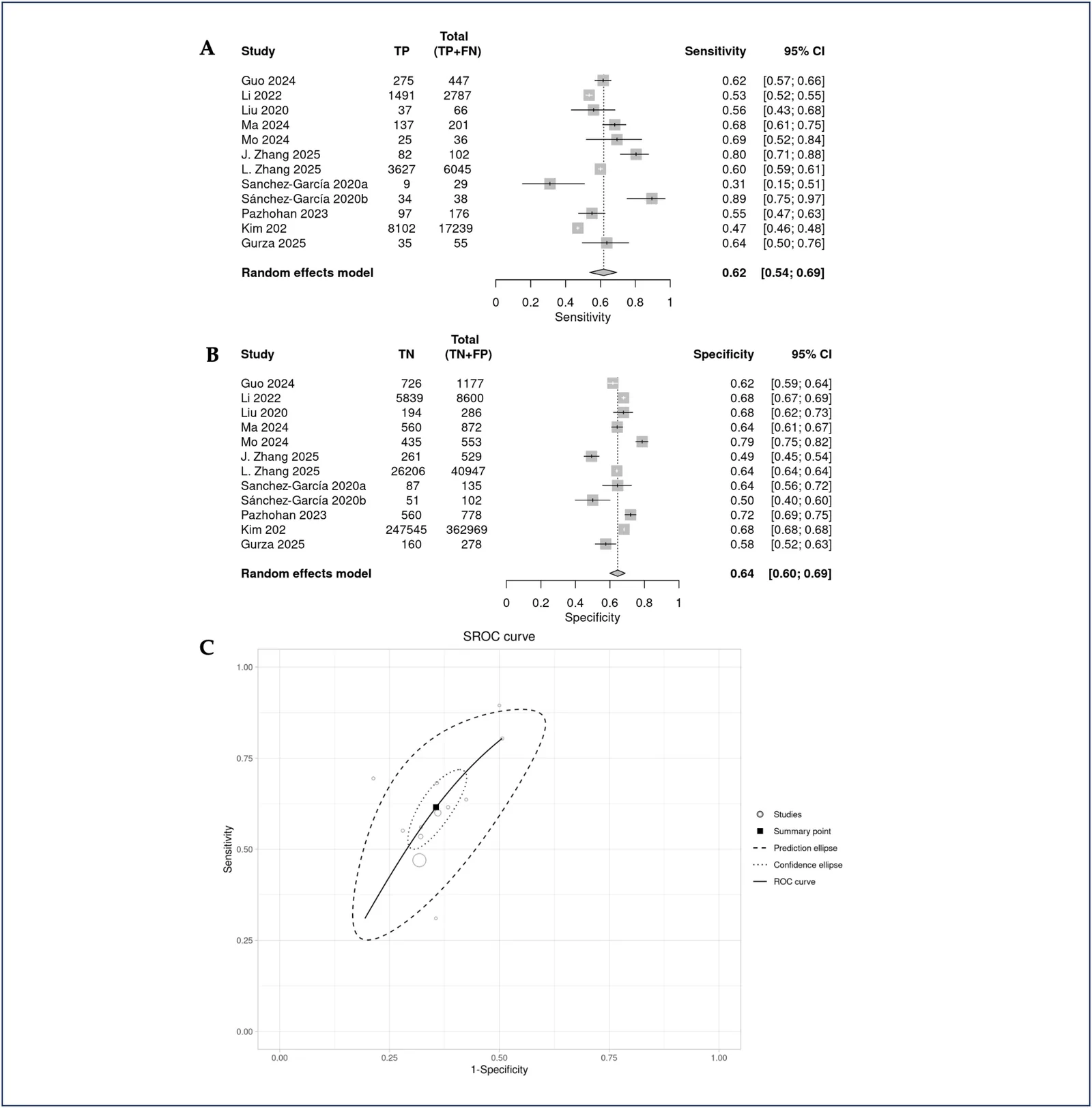
Forest plots showing the pooled sensitivity (A), pooled specificity (B), and the summary receiver operating characteristic (sROC) curve (C) for the predictive performance of elevated TyG in identifying pregnant women at risk of developing GDM

### GRADE assessment and Funnel plot

We assessed the certainty of the evidence for the association between an elevated TyG index in the first trimester and the risk of developing GDM using the GRADE approach. Our analysis included 13 non-randomized studies involving pregnant women with TyG index measurements in early pregnancy (first trimester). Chart 3 shows a 140% increased risk of developing GDM associated with an elevated TyG index in the first trimester of pregnancy, as well as a 2.4-fold higher likelihood of developing GDM compared to pregnant women with normal TyG index values (OR: 2.39; 95% CI: 1.94–2.93). According to GRADE, the certainty of the evidence was rated as moderate. These findings suggest that the TyG index may serve as a useful marker for identifying pregnant women at higher risk of GDM. However, further high-quality studies are needed to confirm this association and strengthen the evidence. No evidence of publication bias was found (Egger's test:^(44)^ 1.67; 95% CI -0.65 to 41.4; p > 0.18), indicating that publication bias was not statistically significant. Overall, these findings suggest that the results of our meta-analysis are not affected by study selection (Figure 5).

**Chart 3:** Certainty of Evidence through GRADE. Pregnant women with elevated TyG in the first trimester have 2.4-fold higher odds of developing GDM compared with those with normal TyG values

Outcomes	№ of participants (studies) Follow-up	Certainty of the evidence (GRADE)	Relative effect (95% CI)
Risk of GDM	(13 non-randomised studies)	⨁⨁⨁◯ Moderate	OR 2.35 (1.88 to 2.94)

CI: confidence interval; OR: odds ratio; Moderate certainty: we are moderately confident in the effect estimate: the true effect is likely to be close to the estimate of the effect, but there is a possibility that it is substantially different

**Figure 5:**
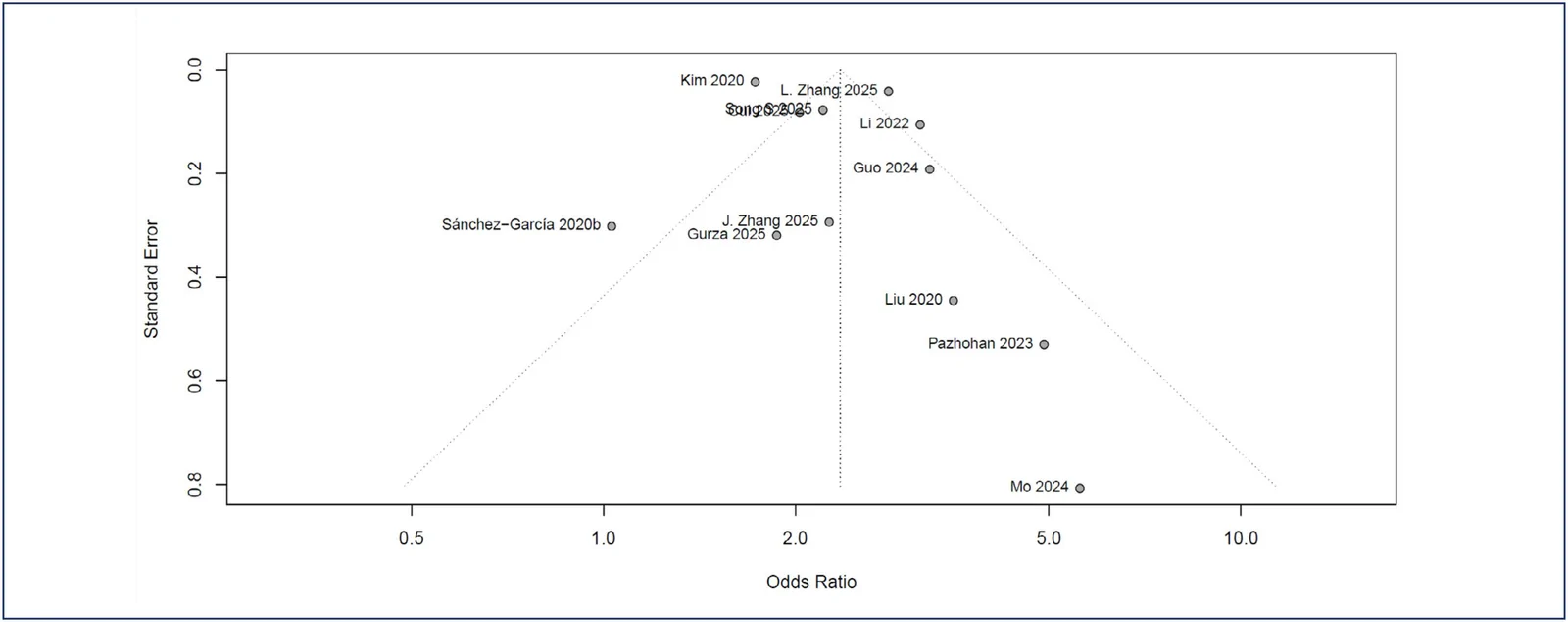
Funnel plot of the studies included in the meta-analysis assessing the association between elevated TyG and the risk of GDM, with Egger's test: 1.74; 95% CI −0.84 to 4.33; p: 0.22. No evidence of publication bias was observed

## Discussion

Our updated meta-analysis of 13 observational studies including a total of 465,655 pregnant women shows that an elevated TyG index in the first trimester is associated with a 137% higher risk of developing gestational GDM (OR 2.37; 95% CI 1.92–2.94), and this association remained robust in sensitivity analyses. In subgroup analyses by region, this increased risk was observed only in Asian populations (OR 2.64; 95% CI 2.21–3.15), with no significant association in non-Asian populations. In addition, each 1-unit increase in the TyG index was associated with a 1.76-fold higher risk of developing GDM.

From a pathophysiological standpoint, GDM encompasses impaired glucose homeostasis emerging from the second trimester of pregnancy, driven by an abnormally increased state of insulin resistance together with an inadequate compensatory pancreatic β-cell response.^(28,45)^ Consequently, pregestational factors such as overweight, obesity, dyslipidemia, as well as other variables including physical inactivity, hypercaloric diets, and components of the metabolic syndrome are closely related to the development of GDM.^(46)^ Traditionally, the pathophysiology of GDM has been attributed primarily to the role of placental lactogen; however, its contribution is relatively modest and mainly involves modulating the response to insulin resistance, which, as noted, is largely determined by factors that are not intrinsic to pregnancy itself. In this context, surrogate markers such as the TyG index, as a proxy for insulin resistance, may serve as early predictors not only of GDM but also of other conditions associated with insulin resistance.^(28)^

To date, two systematic reviews and meta-analyses (SR-MAs) have evaluated the association between the TyG index and GDM; however, both were conducted more than five years ago and included at most five primary studies.

Liu Y et al.^(12)^ conducted a meta-analysis including only four observational studies to assess the performance of the TyG index as a biomarker for GDM. In that study, TyG was analyzed exclusively as a continuous variable using meta-analysis of mean differences (MDs), rather than ORs, between the exposed and unexposed cohorts. The authors reported that the pooled MD for TyG was significantly higher in the GDM group compared with controls (MD 0.22; 95% CI 0.07–0.36; I^2^ = 71%). However, this difference was no longer evident in subgroup analyses restricted to Asian studies (MD 0.45; 95% CI −0.10 to 1.01; I^2^ = 45%) or non-Asian studies (MD 0.16; 95% CI −0.02 to 0.34; I^2^ = 75%). They also performed subgroup analyses according to the gestational age at TyG assessment (first vs second trimester), reporting that TyG measured in the second trimester (MD 0.26; 95% CI 0.15–0.37; I^2^ = 71%) showed significant differences, whereas TyG measured in the first trimester did not (MD 0.22; 95% CI −0.07 to 0.51; I^2^ = 84%). Nonetheless, the studies interpreted as evaluating TyG in the second trimester corresponded to two investigations by Sánchez-García et al.^(38,39)^ in Mexican pregnant women; in one of these, TyG values in both trimesters were directly compared, and no association was found between first-trimester TyG and the development of GDM (RR 1.03; 95% CI 0.57–1.88; p = 0.90). We therefore consider this trimester-based subgroup analysis to be flawed, as both studies actually evaluated TyG in the first trimester rather than the second. Moreover, the meta-analysis did not adequately explore the sources of the substantial between-study heterogeneity, which further downgrades the overall quality of the evidence from a methodological standpoint.

Conversely, Song et al.^(13)^ conducted a systematic review and meta-analysis including five observational studies, with the same overarching objective as the previously described meta-analysis: to evaluate the TyG index as a predictor of GDM. In their pooled analysis, they reported an overall increased risk of GDM associated with elevated TyG (OR 2.52; 95% CI 1.33 to 4.76; I^2^ = 65%), and this association remained consistent in subgroup analyses stratified by study design (prospective vs retrospective), accompanied by a reduction in heterogeneity. When they stratified by region (Asian vs non-Asian pregnant women), no significant association was observed among non-Asian women (OR 0.96; 95% CI 0.35–2.63). These findings are broadly consistent with ours; however, our updated SR-MA includes a total of 15 primary studies, thereby providing more robust evidence supporting the association between elevated TyG and the risk of GDM. From a methodological perspective, Song et al. assessed risk of bias using the Newcastle–Ottawa Scale (NOS), rating all included studies as having low risk of bias. In contrast, we used the more granular ROBINS-E tool, which allowed us to identify that some of these studies were actually at "some concerns" risk of bias (Pazhohan et al.^(40)^) and at "very high" risk of bias (Sánchez-García et al.^(39)^).

It is important to emphasize that our study incorporates a detailed assessment of heterogeneity, including *Influence Analysis* and *GOSH* plots to detect outliers. In addition, we performed meta-regression using clinically relevant covariates such as risk of bias and patient origin by region (Asian vs non-Asian), which, although they explain only about 20% of the between-study heterogeneity, provide a basis for future research on the role of geographic origin.

In our subgroup analyses, we found that the association between elevated TyG and the risk of GDM was significant only in Asian populations, whereas in non-Asian women the effect size was smaller and did not reach statistical significance. This pattern is consistent with previous meta-analyses in which TyG has been described as a predictor of GDM primarily in Asian women, but not in women from other ethnic backgrounds.^(13,47)^ The greater susceptibility of Asian women to develop GDM at lower BMI levels, together with a higher degree of visceral adiposity for a given body mass index and a reduced pancreatic β-cell compensatory capacity, may amplify the contribution of insulin resistance to the pathophysiology of GDM in these populations, thereby increasing the discriminatory value of a glucose- and triglyceride-based index such as TyG.^(39,48)^ Conversely, in non-Asian populations, the smaller number of available studies, differences in GDM diagnostic criteria, and the greater prominence of other metabolic determinants (overt obesity and lifestyle factors) may account for the lack of statistically significant associations, rather than indicating a true absence of an underlying biological relationship.

Our study demonstrates relevant strengths. First, it includes a sample size exceeding 465,000 pregnant women, thereby enhancing its population representativeness. We performed an extensive and methodologically stringent search across multiple databases, aligned with PRISMA, Cochrane, and AMSTAR 2 guidelines and supported by robust statistical analyses. The risk of bias was rigorously assessed using ROBINS-E, and the certainty of the evidence was evaluated with GRADE approach, further reinforcing the methodological rigor of the review. Our results demonstrate robustness and reliability, supported by a detailed assessment of heterogeneity, the identification of outliers, and consistent results across the included studies. Moreover, we also evaluated diagnostic accuracy, including sensitivity, specificity, likelihood ratios, and the AUC, which complemented the association analyses. Additionally, we conducted subgroup analysis to identify differences according to ethnicity.

This study also exhibits several limitations. The exclusively observational design of the included studies does not permit the establishment of causal inferences and reveals substantial variability in confounder adjustment, considering that two of the included studies did not perform any confounder adjustment. The predominance of research conducted in Asian populations may limit the global generalizability of the findings to other ethnic and demographic groups. Furthermore, the absence of a standardized cutoff value for the TyG index makes it difficult to provide uniform clinical recommendations. Considerable heterogeneity was also observed among the studies, likely attributable to epidemiological and clinical differences between populations, variability in TyG thresholds, discrepancies in measurement methods and differing levels of confounder adjustment, among other factors. Finally, although no evidence of publication bias was detected, the certainty of the evidence was rated as moderate, highlighting the need for more higher-quality research, particularly well-designed prospective studies, to enhance the robustness and applicability of these findings.

## Conclusion

Our study suggests that the TyG index, as a surrogate marker of insulin resistance, is associated with an increased risk of GDM when elevated during the first trimester. An elevated TyG index was associated with a 137% higher likelihood of developing GDM, supported by a moderate level of evidence. Given the limited literature on early-pregnancy identification of GDM, these findings provide a relevant basis for future research. While the TyG index may have potential value as an early risk marker, its performance as a screening tool should be interpreted cautiously, and further robust studies are required before clinical application can be established.

## Data Availability

The research data are described in the article presented.
