# Supplementary material for: Association between triglyceride–glucose index (TyG) as a surrogate marker of insulin resistance and risk of gestational diabetes mellitus (GDM): updated systematic review and meta-analysis
Source: Rev Bras Ginecol Obstet. 2026 Oct 13;48:e-rbgo47. doi: 10.61622/rbgo/2026rbgo47 (PMC13690370; doi:10.61622/rbgo/2026rbgo47)
Supplement: Supplementary file 1 [file 1806-9339-rbgo-48-e-rbgo47-Supp01.pdf]

Supplementary material

Table 1S. Search strategy

		Results	Date
**1. MEDLINE VIA PUBMED**			
#1	"triglyceride glucose index"[tiab] OR "triglyceride-glucose index"[tiab] OR "TyG index"[tiab] OR TyG[tiab])	3062	August 12, 2025
#2	"Diabetes, Gestational"[Mesh] OR ("gestational diabetes"[tiab] OR "gestational diabetes mellitus"[tiab] OR GDM[tiab])	31221	
#3	#1 AND #2	51	
2. SCOPUS			August 12, 2025
#1	TITLE-ABS-KEY (triglyceride glucose INDEX OR triglyceride-glucose INDEX OR tyg INDEX)	28285	
#2	TITLE-ABS-KEY (diabetes gestational)	48723	
#3	TITLE (#1 AND #2)	31	
3. EMBASE			August 12, 2025
#1	('triglyceride glucose index':ab,ti OR 'triglyceride-glucose index':ab,ti OR 'tyg index':ab,ti OR tyg:ab,ti)	3654	
#2	'gestational diabetes mellitus'/exp OR ('gestational diabetes':ab,ti OR gdm:ab,ti)	66605	
#3	#1 AND #2	78	
4. Web of Science			August 12, 2025
#1	("triglyceride glucose index" OR "triglyceride-glucose index" OR "TyG index" OR (tyg NEAR/1 index) (All fields)	2916	
#2	("gestational diabetes" OR GDM) AND (pregnan* OR gestation) (All fields)	32871	
#4	#1 AND #2 AND #3	134	
5. Sciencedirect			August 12, 2025
#1	("triglyceride glucose index" OR "triglyceride-glucose index" OR "TyG index")	412	
#2	("gestational diabetes" OR GDM)	8795	
#3	#1 AND #2	1	

Table 2S. Excluded studies

ID	Author	Study title.
1	Al-Rawi H et al. 2024	The utility of maternal adiponectin and triglyceride-glycemic index for gestational diabetes mellitus screening: a cross-sectional study
2	Bai X. et al. 2024	Second-trimester triglyceride-glucose index to predict adverse outcomes in women with gestational diabetes mellitus: a retrospective multicenter cohort study
3	Bugday I. et al. 2024	The relationship between coronary reserve and the TyG index in patients with gestational diabetes mellitus.
4	Chen X. et al. 2024	Characteristics of glucose-lipid metabolism in early pregnancy among overweight and obese women in their predictive value for gestational diabetes mellitus.
5	Dinakaran A. et al. 2025	Prediction Models for Gestational Diabetes Mellitus: Diagnostic Utility of Clinical and Biochemical Markers.
6	Duo Y. et al.	The predictive performance of triglyceride and triglyceride-glucose index in the first trimester for gestational diabetes mellitus: a prospective cohort study
7	Genova M. et al. 2022	Association between pre-pregnancy body mass index and triglyceride glycemic index in mid pregnancy in women with gestational diabetes.
8	Guo Y. et al. 2024	Triglyceride-glucose index in early pregnancy predicts the risk of gestational diabetes: a prospective a cohort study
9	Khan S. et al. 2022	Utility of TyG index in gestational diabetes mellitus
10	Liang X. et al. 2025	Association between triglyceride glucose-body mass index and gestational diabetes mellitus: a prospective cohort study.
11	Pan Y. et al. 2023	Is there any association between early trimester Triglyceride–glucose index and incidence of hypertensive disorder of pregnancy and adverse pregnancy outcomes?
12	Pari N. et al. 2023	Triglyceride glucose index as a surrogate marker of insulin resistance and its correlation with serum leptin levels in gestational diabetes.
13	Rakchna M. et al. 2021	Atherogenic lipid indices in gestational diabetes mellitus-to worry or not to worry?
14	Song G. et al. 2024	The predictive ability of the triglyceride glucose index, fasting glucose and oral glucose tolerance test for postpartum hyperglycemia in women with a GDM history.
15	Tang L. et al. 2018	Association of lipid profiles and triglyceride-glucose index at the first visit with the risk of gestational diabetes mellitus
16	Tankasali M. et al. 2025	Correlation of triglyceride glucose index and serum uric acid levels in early pregnancy (<20-week period of gestation) with the development of gestational diabetes mellitus.
17	Xiao Y et al. 2024	Relationship between serum Hcy, mannose-binding lectin, TyG index, insulin resistance and adverse pregnancy outcomes in patients with gestational diabetes mellitus during the second trimester.
18	Yang X. et al. 2024	Association of Triglyceride to High Density Lipoprotein-Cholesterol Ratio In Early Pregnancy with the Risk of Gestational Diabetes Mellitus: Large-sample Retrospective Cohort Study.
19	You Y. et al. 2023	Association between the triglyceride to high-density lipoprotein cholesterol ratio and the risk of gestational diabetes mellitus: a second analysis based on data from a prospective cohort study
20	Yuan X. et al. 2025	Relationship between early pregnancy triglyceride-glucose index and gestational diabetes mellitus in twin pregnancies.
21	Zhang Jinghua. et al. 2024	A promising biomarker for predicting risks of adverse pregnancy outcomes in Hangzhou, China. Triglyceride-glucose index: A promising biomarker for predicting risks of<br>adverse pregnancy outcomes in Hangzhou, China
22	Zhao D. et al. 2024	Triglyceride-glycaemic index: Insights into predicting fetal macrosomia and its interaction with gestational diabetes mellitus: A cohort study of Chinese pregnant women
23	Zeng Y et al. 2023	Association of triglycerideglucose index levels with gestational diabetes mellitus in the US pregnant women: a cross-sectional study

Table 3S. Meta-regression

- Variables: CONTINENT+CUTOFF:

Mixed-Effects Model (k = 13; tau^2 estimator: REML)

tau^2 (estimated amount of residual heterogeneity): 0.0507 (SE = 0.0383)
tau (square root of estimated tau^2 value): 0.2252
I^2 (residual heterogeneity / unaccounted variability): 84.10%
H^2 (unaccounted variability / sampling variability): 6.29
R^2 (amount of heterogeneity accounted for): 21.02%

Test for Residual Heterogeneity:
QE(df = 10) = 87.3635, p-val < .0001

Test of Moderators (coefficients 2:3):
F(df1 = 2, df2 = 10) = 2.9012, p-val = 0.1015

Model Results:

	estimate	se	tval	df	pval	ci.lb	ci.ub	
intrcpt	-3.8686	2.8684	-1.3487	10	0.2072	-10.2599	2.5226	
continentNo Asia	-0.5814	0.2691	-2.1608	10	0.0560	-1.1809	0.0181	.
cutoff	0.5645	0.3384	1.6680	10	0.1263	-0.1896	1.3186	

- VARIABLE: CONTINENT:

Mixed-Effects Model (k = 13; tau^2 estimator: REML)

tau^2 (estimated amount of residual heterogeneity): 0.0556 (SE = 0.0382)
tau (square root of estimated tau^2 value): 0.2357
I^2 (residual heterogeneity / unaccounted variability): 88.62%
H^2 (unaccounted variability / sampling variability): 8.79
R^2 (amount of heterogeneity accounted for): 13.49%

Test for Residual Heterogeneity:
QE(df = 11) = 137.3213, p-val < .0001

Test of Moderators (coefficient 2):
F(df1 = 1, df2 = 11) = 2.6386, p-val = 0.1326

Model Results:

	estimate	se	tval	df	pval	ci.lb	ci.ub	
intrcpt	0.9174	0.0969	9.4721	11	<.0001	0.7043	1.1306	***
continentNo Asia	-0.4464	0.2748	-1.6244	11	0.1326	-1.0512	0.1585	

- VARIABLE: CUTOFF

Mixed-Effects Model (k = 13; tau^2 estimator: REML)

tau^2 (estimated amount of residual heterogeneity): 0.0667 (SE = 0.0465)
tau (square root of estimated tau^2 value): 0.2582
I^2 (residual heterogeneity / unaccounted variability): 86.57%
H^2 (unaccounted variability / sampling variability): 7.45
R^2 (amount of heterogeneity accounted for): 0.00%

Test for Residual Heterogeneity:
QE(df = 11) = 95.6185, p-val < .0001

Test of Moderators (coefficient 2):
F(df1 = 1, df2 = 11) = 0.8311, p-val = 0.3815

Model Results:

	estimate	se	tval	df	pval	ci.lb	ci.ub
intrcpt	-2.0815	3.2340	-0.6436	11	0.5330	-9.1994	5.0364
cutoff	0.3464	0.3799	0.9116	11	0.3815	-0.4899	1.1826

- YEAR:

Mixed-Effects Model (k = 13; tau^2 estimator: REML)

tau^2 (estimated amount of residual heterogeneity): 0.0631 (SE = 0.0454)
tau (square root of estimated tau^2 value): 0.2512
I^2 (residual heterogeneity / unaccounted variability): 79.97%

H^2 (unaccounted variability / sampling variability): 4.99
R^2 (amount of heterogeneity accounted for): 1.75%

Test for Residual Heterogeneity:
QE(df = 11) = 46.6470, p-val < .0001

Test of Moderators (coefficient 2):
F(df1 = 1, df2 = 11) = 0.9374, p-val = 0.3537

Model Results:

	estimate	se	tval	df	pval	ci.lb	ci.ub
intrcpt	-93.0278	96.9757	-0.9593	11	0.3580	-306.4698	120.4142
year	0.0464	0.0479	0.9682	11	0.3537	-0.0591	0.1519

- BIAS:
Mixed-Effects Model (k = 13; tau^2 estimator: REML)

tau^2 (estimated amount of residual heterogeneity): 0.0617 (SE = 0.0417)
tau (square root of estimated tau^2 value): 0.2484
I^2 (residual heterogeneity / unaccounted variability): 89.64%
H^2 (unaccounted variability / sampling variability): 9.65
R^2 (amount of heterogeneity accounted for): 3.89%

Test for Residual Heterogeneity:
QE(df = 11) = 138.6897, p-val < .0001

Test of Moderators (coefficient 2):
F(df1 = 1, df2 = 11) = 0.7953, p-val = 0.3916

Model Results:

	estimate	se	tval	df	pval	ci.lb	ci.ub	
intrcpt	0.6583	0.2509	2.6241	11	0.0237	0.1061	1.2104	*
biaslow	0.2434	0.2729	0.8918	11	0.3916	-0.3573	0.8440	
